# The Chevrel phase In_4.07_Mo_15_S_11.87_Se_7.13_ with mixed chalcogenide occupancy

**DOI:** 10.1107/S1600536809007351

**Published:** 2009-03-06

**Authors:** You-Soon Lee, Sung Kwon Kang

**Affiliations:** a Department of Chemistry, Chungnam National University, Daejeon 305-764, Republic of Korea

## Abstract

The single-crystal of the title compound, indium pentadecamolybdenum nonadeca(sulfide/selenide), was obtained by solid state reaction with an S/Se mixture. It adopts the structure type of In_3_Mo_15_Se_19_ and In_3.7_Mo_15_S_19_, which are non-substituted Chevrel phases in the space group *P*6_3_/*m*. The Mo, one S/Se and two In sites have point symmetry *m*.. and two S/Se and one In atoms are in 3.. sites. This compound contains isolated Mo_6_ and Mo_9_ clusters. The shapes of clusters are octa­hedral and confacial biocta­hedral, respectively, face-capped by chalcogen atoms over each triangle face. The Mo—*X* bonds (*X *= S, Se) play an important role for the constitution of the framework. The Mo—*X* distances of 2.479 (2)–2.6687 (9) Å are within the ranges of average values of Mo—S and Mo—Se distances. The In atoms located on sites with *m*.. symmetry are partially occupied.

## Related literature

For discussion of the crystal structures of Chevrel phases, see: Grüttner *et al.* (1979 ▶). For applications, see: Suresh *et al.* (2008 ▶); Aurbach *et al.* (2007 ▶). For the syntheses and crystal structures of Chevrel phases with various cations, see: Salloum, Gautier *et al.* (2004 ▶); Salloum, Gougeon *et al.* (2004 ▶).

## Experimental

### 

#### Crystal data

In_4.07_Mo_15_S_11.87_Se_7.13_
*M* *_r_* = 2847.4
Hexagonal, 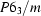

*a* = 9.5974 (2) Å
*c* = 19.1668 (5) Å
*V* = 1528.93 (6) Å^3^
*Z* = 2
Mo *K*α radiation
μ = 18.17 mm^−1^
*T* = 295 K
0.04 × 0.04 × 0.03 mm

#### Data collection

Bruker SMART CCD area-detector diffractometer
Absorption correction: multi-scan (*SADABS*; Bruker, 2002 ▶) *T*_min_ = 0.431, *T*_max_ = 0.577
10366 measured reflections
1309 independent reflections
1047 reflections with *I* > 2σ(*I*)
*R*_int_ = 0.048

#### Refinement

*R*[*F* ^2^ > 2σ(*F* ^2^)] = 0.039
*wR*(*F* ^2^) = 0.090
*S* = 1.41
1309 reflections
77 parameters
Δρ_max_ = 4.14 e Å^−3^
Δρ_min_ = −4.12 e Å^−3^

### 

Data collection: *SMART* (Bruker, 2002 ▶); cell refinement: *SAINT* (Bruker, 2002 ▶); data reduction: *SAINT*; program(s) used to solve structure: *SHELXS97* (Sheldrick, 2008 ▶); program(s) used to refine structure: *SHELXL97* (Sheldrick, 2008 ▶); molecular graphics: *DIAMOND* (Brandenburg, 1998 ▶); software used to prepare material for publication: *WinGX* publication routines (Farrugia, 1999 ▶).

## Supplementary Material

Crystal structure: contains datablocks global, I. DOI: 10.1107/S1600536809007351/br2099sup1.cif

Structure factors: contains datablocks I. DOI: 10.1107/S1600536809007351/br2099Isup2.hkl

Additional supplementary materials:  crystallographic information; 3D view; checkCIF report

## Figures and Tables

**Fig. 1.**
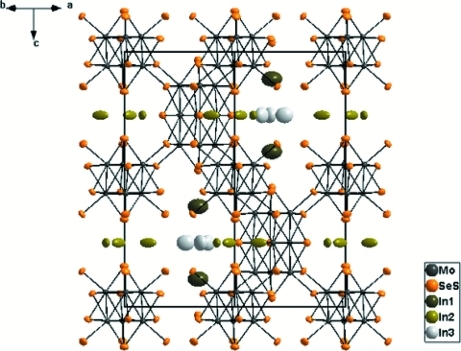
View of the crystal structure of the title compound along [110], with displacement ellipsoids at the 80% probability level.

**Fig. 2.**
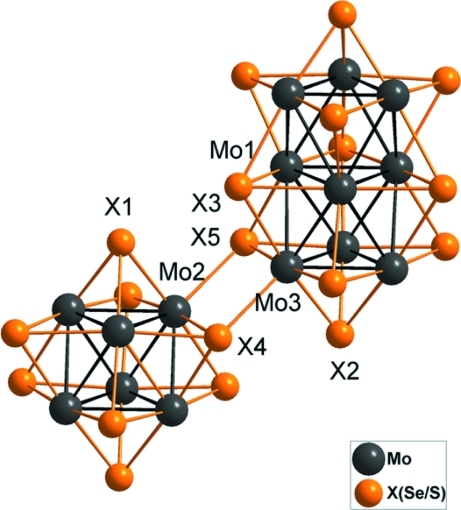
Mo6X8 and Mo9X11 cluster units interconnected through Mo—X bonds (X=Se/S).

## supplementary crystallographic information

### Comment

The classical Chevrel phases, containing blocks of Mo_6_*X*_8_, have been in interest for both structural respects and application to rechargeable batteries (Suresh, *et al.*, 2008; Aurbach, *et al.*, 2007). The new Chevrel phases In*_x_*Mo_15_Se_19_ (*x*=2.9 and 3.3) also have been studied by X-ray single-crystal method (Grüttner *et al.*, 1979). These were the first compound having a transition metal cluster with the isolated Mo_6_ and Mo_9_ clusters. The Mo_9_ cluster has the shape of a confacial bioctahedron resulting from the condensation of two octahedral Mo_6_ clusters. Both clusters are surrounded by face-capping Se atoms to form Mo_6_Se_8_ and Mo_9_Se_11_ cluster units, and they are interconnected through Mo—Se bonds to build the three dimensional framework (Fig. 1). On our continuous studies to develop new materials for rechargeable batteries, herein, we report the single-crystal structure of the mixed chalcogenide compound In_4.07_Mo_15_S_11.87_Se_7.13_ (1). We have investigated the effect of the partial substitution of Se by S atoms in the related Chevrel phase, hoping that the building blocks of Chevrel phase would not be changed.

The crystal structure of the title compound in a unit cell is shown in Fig. 1. The framework is composed of Mo_6_*X*_8_ and Mo_9_*X*_11_ cluster units (*X*=Se/S) that are interconnected through Mo—*X* bonds. The Mo_6_ cluster forms the octahedral geometry with Mo—Mo bonds between the six Mo atoms, and the eight faces on the octahedron share a chalcogen atom to create the Mo_6_*X*_8_ building block (Fig. 2). The Mo_9_ cluster is formed by one dimensional *trans*-face sharing of two Mo_6_ octahedron, and surrounded by eleven face-capping chalcogen atoms. The Mo—Mo bond distance related through the threefold axis in the Mo_6_ clusters is 2.6728 (11) Å. And the Mo—Mo distances within the Mo_9_ clusters are in the range of 2.6415 (10) - 2.7540 (8) Å which are within the normal range of the other Chevrel phases (Grüttner *et al.*, 1979; Salloum, Gautier *et al.*, 2004; Salloum, Gougeon *et al.*, 2004). The amount of substitution of Se atoms by S atoms are dependent on the atomic positions with the range of 34% (for X3 atom) - 86% (for X1 atom). The higher the S atom occupation, the shorter Mo—*X* bond distances are.

### Experimental

The title compound was prepared from powder elemental indium (99.999 at.%), molybdenum (99.999 at.%), sulfur (99.98 at.%), and selenium (99.99 at.%) from Aldrich products in the slightly off-stoichiometric 5:15:12:7 ratio. The reaction mixture was sealed under a nitrogen atmosphere in a silica tube and heated at 1343 K for 72 h and cooled to room temperature at the rate of 10 K/h to obtain black single crystals for X-ray studies.

### Refinement

The crystal structure of the title compound was solved and refined starting from the atomic coordinates reported for In_~3_Mo_15_Se_19_ compound (Grüttner *et al.*, 1979). In the first stage of the refinement, the positions of all atoms but In3 were obtained reasonably. The remaining In3 atom was located in subsequent difference Fourier syntheses. The maximum and minimum residual electron density peaks were located at 1.07 and 0.46 Å, respectively, from the In1 atom.

### Crystal data

**Table:**

In_4.07_Mo_15_S_11.87_Se_7.13_	*D*_x_ = 6.185 Mg m^−^^3^
*M**_r_* = 2847.4	Mo *K*α radiation, λ = 0.71073 Å
Hexagonal, *P*6_3_/*m*	Cell parameters from 1691 reflections
Hall symbol: -P 6c	θ = 2.5–28.3°
*a* = 9.5974 (2) Å	µ = 18.17 mm^−^^1^
*c* = 19.1668 (5) Å	*T* = 295 K
*V* = 1528.93 (6) Å^3^	Block, black
*Z* = 2	0.04 × 0.04 × 0.03 mm
*F*(000) = 2524.1	

### Data collection

**Table:**

Bruker SMART CCD area-detector diffractometer	1047 reflections with *I* > 2σ(*I*)
φ and ω scans	*R*_int_ = 0.048
Absorption correction: multi-scan (*SADABS*; Bruker, 2002)	θ_max_ = 28.3°, θ_min_ = 2.1°
*T*_min_ = 0.431, *T*_max_ = 0.577	*h* = −12→12
10366 measured reflections	*k* = −9→12
1309 independent reflections	*l* = −25→25

### Refinement

**Table:**

Refinement on *F*^2^	77 parameters
Least-squares matrix: full	0 restraints
*R*[*F*^2^ > 2σ(*F*^2^)] = 0.039	*w* = 1/[σ^2^(*F*_o_^2^) + (0.0327*P*)^2^] where *P* = (*F*_o_^2^ + 2*F*_c_^2^)/3
*wR*(*F*^2^) = 0.090	(Δ/σ)_max_ < 0.001
*S* = 1.41	Δρ_max_ = 4.14 e Å^−^^3^
1309 reflections	Δρ_min_ = −4.12 e Å^−^^3^

### Special details

**Table:**

Geometry. All e.s.d.'s (except the e.s.d. in the dihedral angle between two l.s. planes) are estimated using the full covariance matrix. The cell e.s.d.'s are taken into account individually in the estimation of e.s.d.'s in distances, angles and torsion angles; correlations between e.s.d.'s in cell parameters are only used when they are defined by crystal symmetry. An approximate (isotropic) treatment of cell e.s.d.'s is used for estimating e.s.d.'s involving l.s. planes.

### Fractional atomic coordinates and isotropic or equivalent isotropic displacement parameters (Å^2^)

**Table:**

	*x*	*y*	*z*	*U*_iso_*/*U*_eq_	Occ. (<1)
Mo1	0.16177 (10)	0.50528 (10)	0.25	0.0084 (2)	
Mo2	0.01379 (7)	0.16724 (7)	0.05730 (3)	0.00754 (18)	
Mo3	0.31869 (7)	0.50095 (7)	0.13306 (3)	0.00801 (18)	
Se1	0	0	0.15855 (13)	0.0164 (9)	0.140 (9)
S1	0	0	0.15855 (13)	0.0164 (9)	0.860 (9)
Se2	0.3333	0.6667	0.03438 (13)	0.0137 (9)	0.142 (9)
S2	0.3333	0.6667	0.03438 (13)	0.0137 (9)	0.858 (9)
Se3	0.31626 (16)	0.34882 (16)	0.25	0.0139 (5)	0.658 (8)
S3	0.31626 (16)	0.34882 (16)	0.25	0.0139 (5)	0.342 (8)
Se4	0.71167 (14)	0.03659 (14)	0.05076 (5)	0.0122 (4)	0.437 (6)
S4	0.71167 (14)	0.03659 (14)	0.05076 (5)	0.0122 (4)	0.563 (6)
Se5	0.01082 (16)	0.38207 (15)	0.13790 (6)	0.0149 (5)	0.328 (6)
S5	0.01082 (16)	0.38207 (15)	0.13790 (6)	0.0149 (5)	0.672 (6)
In1	0.6667	0.3333	0.10758 (9)	0.0837 (6)	
In2	0.2155 (3)	0.0510 (3)	0.25	0.0331 (8)	0.468 (4)
In3	0.5545 (8)	0.2420 (7)	0.25	0.055 (2)	0.224 (4)

### Atomic displacement parameters (Å^2^)

**Table:**

	*U*^11^	*U*^22^	*U*^33^	*U*^12^	*U*^13^	*U*^23^
Mo1	0.0086 (4)	0.0086 (4)	0.0080 (4)	0.0043 (4)	0	0
Mo2	0.0079 (3)	0.0083 (3)	0.0066 (3)	0.0041 (3)	−0.0002 (2)	−0.0006 (2)
Mo3	0.0083 (3)	0.0085 (3)	0.0072 (3)	0.0042 (3)	−0.0002 (2)	−0.0005 (2)
Se1	0.0198 (11)	0.0198 (11)	0.0098 (13)	0.0099 (6)	0	0
S1	0.0198 (11)	0.0198 (11)	0.0098 (13)	0.0099 (6)	0	0
Se2	0.0132 (10)	0.0132 (10)	0.0146 (14)	0.0066 (5)	0	0
S2	0.0132 (10)	0.0132 (10)	0.0146 (14)	0.0066 (5)	0	0
Se3	0.0143 (8)	0.0136 (8)	0.0129 (7)	0.0064 (6)	0	0
S3	0.0143 (8)	0.0136 (8)	0.0129 (7)	0.0064 (6)	0	0
Se4	0.0108 (6)	0.0127 (6)	0.0107 (6)	0.0041 (5)	0.0031 (4)	0.0006 (4)
S4	0.0108 (6)	0.0127 (6)	0.0107 (6)	0.0041 (5)	0.0031 (4)	0.0006 (4)
Se5	0.0197 (8)	0.0110 (7)	0.0136 (7)	0.0073 (6)	−0.0028 (5)	−0.0045 (5)
S5	0.0197 (8)	0.0110 (7)	0.0136 (7)	0.0073 (6)	−0.0028 (5)	−0.0045 (5)
In1	0.0967 (10)	0.0967 (10)	0.0579 (10)	0.0483 (5)	0	0
In2	0.0608 (17)	0.0434 (14)	0.0201 (10)	0.0446 (13)	0	0
In3	0.072 (4)	0.043 (4)	0.060 (4)	0.037 (3)	0	0

### Geometric parameters (Å, °)

**Table:**

Mo1—Mo3	2.7123 (7)	Mo3—S3	2.6687 (9)
Mo1—Mo3^i^	2.7540 (8)	Mo3—S4^vii^	2.6034 (12)
Mo2—Mo2^ii^	2.6728 (11)	Mo3—S5^iii^	2.4976 (14)
Mo3—Mo3^iii^	2.6415 (10)	Mo3—S5	2.5827 (15)
Mo1—S3	2.5844 (16)	In1—In3	2.904 (3)
Mo1—S3^i^	2.5681 (16)	In2—In3	2.825 (7)
Mo1—S5^iv^	2.5299 (13)	In2—Mo1^viii^	2.862 (2)
Mo2—S1	2.479 (2)	In1—S2^ix^	2.721 (3)
Mo2—S4^v^	2.5093 (12)	In2—S1^iv^	2.564 (2)
Mo2—S4^vi^	2.5219 (13)	In2—S3	2.518 (3)
Mo2—S4^vii^	2.6101 (13)	In2—S5^viii^	2.8445 (18)
Mo2—S5	2.5880 (13)	In3—S3	2.937 (6)
Mo3—S2	2.430 (2)	In3—S5^viii^	3.055 (4)
			
Mo2^ii^—Mo2—Mo2^viii^	60.0	S1—Mo2—Mo2^ii^	57.39 (3)
Mo3^iii^—Mo3—Mo3^i^	60.0	S4^v^—Mo2—Mo2^ii^	120.33 (3)
Mo3—Mo1—Mo3^iv^	111.46 (4)	S4^vi^—Mo2—Mo2^ii^	60.24 (4)
Mo3—Mo1—Mo3^i^	57.79 (2)	S4^vii^—Mo2—Mo2^ii^	116.94 (3)
Mo3^x^—Mo1—Mo3^i^	108.95 (4)	S4^v^—Mo2—Mo2^viii^	118.11 (3)
Mo3—Mo1—Mo3^x^	144.30 (4)	S4^vi^—Mo2—Mo2^viii^	120.16 (4)
S5—Mo1—S5^iv^	116.27 (7)	S4^vii^—Mo2—Mo2^viii^	57.01 (3)
S5—Mo1—S3^i^	87.54 (4)	S5—Mo2—Mo2^ii^	131.12 (4)
S5—Mo1—S3	95.12 (4)	S5—Mo2—Mo2^viii^	136.62 (4)
S3^i^—Mo1—S3	174.93 (5)	S2—Mo3—S3	173.60 (5)
S5—Mo1—Mo3	58.91 (3)	S2—Mo3—S5^iii^	91.81 (3)
S5^iv^—Mo1—Mo3	152.53 (5)	S5^iii^—Mo3—S5	175.03 (5)
S3^i^—Mo1—Mo3	117.85 (3)	S5^iii^—Mo3—S3	86.03 (4)
S3—Mo1—Mo3	60.45 (2)	S5^iii^—Mo3—S4^vii^	86.33 (4)
S5—Mo1—Mo3^x^	145.74 (5)	S5—Mo3—S4^vii^	98.34 (4)
S5^iv^—Mo1—Mo3^x^	56.23 (3)	S2—Mo3—Mo3^iii^	57.07 (3)
S3^i^—Mo1—Mo3^x^	60.07 (2)	S3^iii^—Mo3—Mo3	116.82 (3)
S3—Mo1—Mo3^x^	118.08 (2)	S3^i^—Mo3—Mo3	119.14 (3)
S5^iv^—Mo1—Mo3^i^	145.74 (5)	S4^vii^—Mo3—Mo3^i^	136.62 (3)
S3^i^—Mo1—Mo3^i^	60.07 (2)	S4^vii^—Mo3—Mo3^iii^	130.06 (4)
S1—Mo2—S4^v^	175.41 (5)	S5^iii^—Mo3—Mo3^iii^	60.26 (4)
S1—Mo2—S4^vi^	92.35 (3)	S5^iii^—Mo3—Mo3^i^	120.21 (4)
S1—Mo2—S4^vii^	90.27 (3)	Mo1^iii^—S3—Mo1	65.07 (5)
S1—Mo2—S5	91.73 (5)	Mo1^iii^—S3—Mo3^iv^	63.42 (3)
S4^v^—Mo2—S5	92.56 (4)	Mo1—S5—Mo3	64.07 (4)
S4^vi^—Mo2—S5	87.58 (4)	Mo2^ii^—S1—Mo2^viii^	65.23 (6)
S4^v^—Mo2—S4^vii^	87.51 (3)	Mo2^xi^—S4—Mo2^xii^	64.49 (4)
S4^vi^—Mo2—S4^vii^	173.73 (5)	Mo2^xi^—S4—Mo3^xiii^	131.31 (5)
S4^v^—Mo2—S4^vi^	89.47 (3)	Mo2^xii^—S4—Mo3^xiii^	127.85 (5)
S5—Mo2—S4^vii^	98.04 (4)		

Symmetry codes: (i) −*x*+*y*, −*x*+1, *z*; (ii) −*y*, *x*−*y*, *z*; (iii) −*y*+1, *x*−*y*+1, *z*; (iv) *x*, *y*, −*z*+1/2; (v) *y*, −*x*+*y*+1, −*z*; (vi) *x*−1, *y*, *z*; (vii) −*x*+*y*+1, −*x*+1, *z*; (viii) −*x*+*y*, −*x*, *z*; (ix) −*x*+1, −*y*+1, −*z*; (x) −*x*+*y*, −*x*+1, −*z*+1/2; (xi) *x*−*y*+1, *x*, −*z*; (xii) *x*+1, *y*, *z*; (xiii) −*y*+1, *x*−*y*, *z*.
